# Practical Tips for using a Human Library approach In medical education

**DOI:** 10.12688/mep.19746.1

**Published:** 2023-10-05

**Authors:** Rebecca Malhi, Grace Perez, Javeria Shafiq, Aaron Johnston

**Affiliations:** 1 Distributed Learning and Rural Initiatives, Cumming School of Medicine, University of Calgary, Calgary, Alberta, T2N 4Z6, Canada

**Keywords:** Human Library, undergraduate medical education, attitudinal change, rural medicine, recruitment, prejudice reduction

## Abstract

A Human Library is a structured event that brings people from different groups together. It simulates the format of a customary library, with ‘Readers’ borrowing ‘Books’, who are human volunteers sharing their lived experiences and perspectives. Rooted in principles of social psychology, Human Libraries provide opportunities for Books and Readers to interact in meaningful dialogue. The goal of each interaction is to give the Reader new understanding of the Book’s life.

The Human Library was originally developed as a strategy to challenge prejudice through conversation and personal connection, but the approach is remarkably versatile. We repurposed it for a medical education context in order to provide learners in medical school with information and inspiration, particularly about rural life and rural medicine. We organized and held two Human Library events where pre-medical and undergraduate medical students (Readers) engaged in dialogue with rural physicians (Books). However, the strategy could be used to address a wide variety of challenging subjects where the potential Readers are biased or lack experience.

This article draws upon research literature and our own experiences of running Human Library events to give practical advice for other organizations who might want to use this novel approach in medical education.

## Introduction

The Human Library (also known as Living Library) is a structured event that brings people from different groups together. It mimics the format of a regular library with readers borrowing books, except that the books are human volunteers who are willing to share personal experiences.

The Human Library concept was originally developed over twenty years ago in Denmark as a strategy to challenge prejudice ( Human Library Organization, 2023). Rooted in principles of social psychology, specifically intergroup contact theory ( Allport, 1954), Human Libraries provide opportunities for ‘Books’ and ‘Readers’ to interact in meaningful dialogue. The goal of each interaction is to give the Reader new understanding of the Book’s life. This can lead to what Allport (1954) described as ‘the perception of common interests and common humanity’, which in turn, can reduce prejudicial attitudes. Research shows that Human Library interactions are effective in increasing knowledge of, and empathy with, members of marginalized groups ( Bagci & Blazhenkova, 2020; Groyecka *et al.*, 2019; Kudo *et al.*, 2011; Orosz *et al.*, 2016).

According to the official website, Human Library events have been organized and held in over 85 countries ( Human Library Organization, 2023). Despite the increasing popularity of such events, published literature about Human Library initiatives is scarce ( Dobreski & Huang, 2016; Kwan, 2020). We decided to repurpose the concept of a Human Library for use in a medical education context.

Our department is part of the medical school at the University of Calgary, Alberta, Canada. We have now organized and conducted two Human Library events (October 2020 and September 2022). As one component of our departmental mandate is to support doctors in rural communities, the initial Human Library aimed to give undergraduate medical students more information about being a rural physician. This was because our own research showed that many students, particularly those of urban origin, have little knowledge of rural life, often relying on generalizations and stereotypes ( Malhi *et al.*, 2019). Such inaccurate or negative beliefs may dissuade medical students from considering rotations in non-metropolitan locations, or from eventual rural practice. We hypothesized that hearing from rural physicians about their experiences would provide medical students with accurate information, and possibly lead to more favourable attitudes towards establishing a practice in a rural area.

The second Human Library was less focused on recruitment of rural doctors. Like a regular library, we decided to hold the event as an ‘Information Commons’. It was more publicly accessible as we invited both pre-medical and undergraduate medical students to attend, and the topics that were discussed were more general.

In both sessions, we recruited a variety of speakers as Books to discuss a particular aspect of their lives or work. Each Book could be ‘checked out’ by interested Readers. In this paper, we draw upon research literature and our own experiences of running Human Library events to provide practical tips for other organizations who might want to use this novel approach in medical education.

## Tip 1: Start planning for a Human Library event well in advance

Planning a Human Library event is similar to planning a mini-conference. Kudo *et al.* (2011) note that their event, held at Dokkyo University, Japan, took six months of preparation. Many decisions will need to be made, including the format of the event, the venue, which Books will be recruited, collating of stories, advertising and social media, etc. Ideally, the planning tasks will be handled by several team members.

Two of the more time-consuming steps included obtaining approval from the university’s research ethics board and applying to the Undergraduate Medical Education (UME) group for access to the students. In addition, we applied for funding for the Human Library. As we mention in Tip #3, events can be held on a small budget, but having funding does allow organizers to offer perks like catering and honoraria. We recommend applying for funding and ethics board approval well in advance of the Human Library event.

## Tip 2: Determine appropriate format for the event (in-person or virtual)

The appropriate format for a Human Library will depend on your goals and the availability of the participants. To date, our department has conducted one virtual and one in-person Human Library at the University of Calgary. Our virtual Human Library was originally scheduled in October 2020 as a face-to-face event. However, due to COVID-19 restrictions, we had to switch it to an online format, hosting it on Zoom. The second Human Library followed the traditional in-person format and was held in September 2022.

Each format has advantages and disadvantages. Holding a virtual Human Library may work better for some organizations as there is less expense for venue rental, accommodations, and catering. It may also be more convenient for Books to attend virtually. For example, some of our Books are busy rural doctors and appreciated not having to travel long distances to the event. However, holding a virtual Human Library does require that the organizers have some technological expertise, as attendees must be seamlessly moved in and out of online breakout rooms. Another potential downside is ‘Zoom fatigue’ ( Riedl, 2022): we found that it was difficult to recruit Readers to attend a virtual event held in the evening after a day filled with online classes. Perhaps most importantly, an online Human Library means that there is no physical presence between the two parties. Without the eye contact or the ability to read subtle non-verbal cues that express interest or empathy, it is more challenging for Readers and Books to make the mutual emotional connection that is at the heart of the Human Library concept.

In our experience, a face-to-face Human Library is more attractive to attendees. Only twelve people registered for our virtual event, while over forty learners signed up for the in-person Human Library. Face-to-face Readers were curious about the process, tempted by the promise of free food, and genuinely interested in learning more about the Books’ stories. Although an in-person Human Library can be more work to organize, we think that it is preferable to a virtual event. Most of the tips in this paper refer to a face-to-face Human Library format.

## Tip 3: Make decisions about event logistics

Some of the major decisions that event organizers must make include:

•   How much will the event cost? Some logistics will depend on the available funding, but Human Libraries can be surprisingly cost-effective. Books may be paid honoraria, or they may volunteer their time. You may decide to cater the event or provide Books with identifying t-shirts, or not. There is also no need to rent a venue, as any area that has sufficient space to have private conversations can hold the event. We applied for, and received, a grant to pilot our Human Library. This allowed us to provide honoraria to the Books, cater the event, and offer reimbursement for travel and accommodation expenses. We were also able to have door prizes and draws for gift cards and university-affiliated merchandise; these were greatly appreciated by the attendees of our in-person event.

•   When to hold the event? Some Human Libraries are constrained by the schedules of their speakers and potential audience. Our events were held on weekday evenings as many of our Books are busy physicians. We also checked with the medical school’s undergraduate students’ office to ensure that the date for the Human Library would not conflict with other mandated events, such as a scheduled exam. If you choose to hold the Human Library outside working hours, ensure that you have sufficient staff to set up and keep the event running smoothly.

•   How many Books to recruit? This will depend on the time frame of the event: how long is the event? How many times will the Book tell their story? Your decision about how many Books to recruit may also depend on your budget, if you will be providing them with honoraria. We recruited nine Books for our virtual event. At our in-person Human Library, we had nine Books (we recruited ten Books, but one Book cancelled on the day of the event), who told their stories three times during the evening.

•   How many Readers per Book? Some Human Library events only have one Reader per Book ( Dreher & Mowbray, 2012), while others allow Book sessions with up to twenty Readers ( Blizzard *et al.*, 2019). Having multiple Readers per Book is practical as it allow more people to hear the story. It can also be less intimidating for Readers, who may be uncomfortable speaking to a stranger about an unfamiliar topic ( Blizzard *et al.*, 2019). A group session may permit richer conversations as there are a variety of perspectives and voices interacting with the Book. However, too many Readers in a group can reduce the intimacy of the interaction. At our in-person Human Library, we had a comfortable ratio of six Readers (maximum) for each Book.

•   How to organize the time? The length of each reading session can vary. Some Human Libraries schedule up to one hour per session ( Blizzard *et al.*, 2019); other sessions are much shorter. Regardless of the length of the session, the Book will speak for most of the time, and then allows the Reader(s) to ask questions and initiate a conversation about the topic. At our virtual Human Library event, Books had 15 minutes to tell their stories. At the second in-person event, we used a 10-10-10 format, with Books speaking for ten minutes, followed by ten minutes for questions, then ten minutes for transitioning to the next session. We found that ten minutes did not give Books sufficient time to discuss their experiences, and we recommend that Books are given at least 15 minutes per session. It helps to have a time-keeper and moderator to keep the transition of Readers between sessions moving smoothly (see Tip #10).

•   How to organize the space? The goal of organizing the physical layout is to create a welcoming space where Books and Readers can safely hold informal, and even potentially intimate, conversations. Each Book should have a separate area, spaced out for privacy, with either a cluster of chairs or a table with chairs. At our in-person event, we set up the physical space like a café: each table was covered with a cloth and featured a copy of the Book’s ‘jacket’ (see Tip #5). Readers finished a session with a Book, and moved to another table for their next session.

## Tip 4: Recruit Books with engaging stories

A typical library contains a selection of curated books, and a Human Library is no exception. Brown (2016) stated that a key element of a successful Human Library event is to ‘compile an eclectic collection of living books’ (p. 4). When deciding on potential Books, we sought credible speakers with relevant narratives that would be of interest to our audience. Another consideration was whether the Book’s unique circumstances were aligned with the purpose of the Human Library event (knowledge dissemination, student motivation, prejudice reduction, etc.).

We found that the most successful Books have certain characteristics. They need to be passionate about their work or life experiences, and be able to tell their stories in a way that engages the Readers. In cases where the topic is sensitive, the Book should be willing and able to share their vulnerability with others, sometimes multiple times during the event.

For both of our Human Library events, we used our professional contacts to identify potential Books and approached them to ask if they would share their stories. We also posted a description of the Human Library in our departmental newsletter and asked for volunteers to be a Book. Posting a short description of a Human Library and an online recruitment form on your department’s website is another way to find potential Books.

It is important to recognize that recruitment can take a long time, especially as the Human Library concept is new to many people. It may be necessary to explain the purpose of the event and potential benefits to the audience before a Book will agree to participate. However, after a series of successful Human Libraries have been held, individuals may contact organizers to volunteer as a Book ( Blizzard *et al.*, 2019).

We suggest that organizers provide potential Books with the event dates being considered and at a later date, confirm with the Books that they are available to participate. It is also a good idea to have a back-up plan in place in case of a last-minute cancellation by a Book. For example, you might want to ask one or more alternate Books to be available on standby. In our case, one Book had an emergency just a few hours before the in-person Human Library and had to pull out of the event. Our back-up plan included how to re-assign their registered Readers equitably to other Books, so that no Book was overwhelmed.

## Tip 5: Curate stories and create a catalogue of attractive book ‘jackets’

Event organizers must not only curate the Books, but also the topics that are discussed. Although not every story is suitable for the Human Library, Books with difficult or uncomfortable narratives should not be automatically excluded. On the contrary, Jackson *et al.* (2015) found that Readers were highly interested in Books who described how they overcame challenging life experiences. As the Reader-Book interaction is short, the topics should be relevant and focused. For example, a Book could talk about a snippet of their experiences, but should not discuss their life history.

Several months before the event, organizers should ask the Books to provide the title and a brief description of their talk. This allows Books to define their topic and also gives organizers the opportunity to weed out irrelevant or duplicate stories. The description should be a short paragraph in length, and written in a first-person narrative style. We provided Books with examples of talk titles and descriptions, and there are many examples from other Human Libraries that are available on the internet. This gave us an opportunity to guide the Books on crafting their take-home message for the Readers.

For our Human Library events, we also requested Books to send us their contact information, a short biography, and a headshot or photograph. We assembled the talk title, talk description, biography, and author’s picture with a representative graphic to create a ‘book jacket’. Together, the book jackets form a ‘catalogue’ of available Books at the Human Library.

## Tip 6: Advertise the Human Library event

Advertising the Human Library is another key component for organizers, and should probably be done in stages. The initial advertisement is basically a ‘save the date’ news item for the target audience, so that they can make plans to attend. As organizers confirm the details regarding the Books and their stories, additional promotional material can be disseminated to potential attendees of the event. Finally, as the event draws closer, the amount of advertising via social media should increase.

There are many low-cost ways to advertise a Human Library event. We uploaded the catalogue of book jackets to our website so that potential Readers could learn about available Books. Information can also be disseminated via physical posters or electronic bulletin boards, newsletters, and social media accounts. As our Human Library events included post-session student evaluations, we had to be mindful that the contents of the advertisements were consistent with constraints set by the ethics review board.

We agree with Blizzard *et al.* (2019) that word of mouth tends to generate the most interest among potential attendees. For our first Human Library, we recruited student members of the rural and family medicine interest groups to spread the word about the event. Similarly, for our second event, we reached out to an affiliate organization with parallel and comparable interests and asked them for support in recruiting Readers. In both cases, the synergy of our joint efforts was the most effective way to reach our audience.

## Tip 7: Register Readers and collect pre-event information

Pre-registration of Readers is necessary for logistical purposes. For example, you may want to determine the level of interest in the event, how much catering is required, or whether another round of advertising should be scheduled.

Registration is also important for the safety of the Books so that unauthorized people do not have access to them. We used an online registration form to confirm the applicant was a university student. The form included an introductory paragraph about the Human Library and learning objectives for the event, followed by questions to collect the potential Reader’s contact information and demographic data (gender, age, year of study, and any other relevant information for organizers). If funding has been obtained for the event, registration data is useful to include in the report to the granting agency on how the funds were used.

The registration form also asked participants about their reasons and expectations for registering for the event, as well as questions for scheduling sessions. Due to the limited time available during an event, a Reader will not be able to listen to every Book. Therefore, based on the title/description from the catalogue of Books (see Tip #5), each Reader was asked to select their top three ‘must read’ Books. Where possible, their preferences were respected and they were scheduled to attend a session with those Books. A second email was sent to Readers with their schedule and a pre-event questionnaire (see Tip #11).

## Tip 8: Prepare Books for session

Most people who are recruited as Books for a Human Library may not be familiar with the concept. At the time of recruitment, we sent our Books a ‘Guidelines for Books’ document ( Malhi *et al.*, 2023) that contained helpful tips for preparing their stories and what to expect at the event. Blizzard *et al.* (2019) also suggest that organizers should help Books to prepare for the Human Library with the following advice: 1) remind the Book that their session is an informal conversation, not a presentation; 2) the Book does not need any additional materials, such as PowerPoint slides or handouts; 3) the Book does not have to answer any question that makes them uncomfortable; and 4) Books may want to jot down brief notes about what they want to say, so that they don’t forget any important points during the session. Organizers may choose to have a brief orientation for Books prior to the Human Library. The orientation could cover introductions to the moderator and volunteers, directions to the bathrooms, recap of guidelines and safety protocols, etc.

## Tip 9: Ensure physical and psychological safety of the Books 

The role of a Book is a challenging one, as they are often disclosing and discussing highly personal experiences multiple times during a Human Library event. As Kwan (2020) point out, Books can be emotionally vulnerable because of the stories themselves or due to insensitive responses by Readers. There are several ways in which event organizers can provide the Books with physical and psychological safety:

•   Our Human Library events were not open to the general public. Only registered Readers with valid university identification had physical access to the Books (Tip #7).

•   At our in-person event, we gave Books a sense of privacy by distancing their tables (Tip #3). This not only avoided overcrowding, but it also prevented Books and Readers at one table from overhearing stories from adjacent tables.

•   To prevent Books from feeling overwhelmed, we limited the audience during each session to a maximum of six Readers. Also, we purposely designed the event format to have the Books share a maximum of three times (Tip #3).

•   Prior to the event, we sent registrants a ‘Rules for Readers’ document ( Malhi *et al*., 2023). These rules requested respectful behaviour when interacting with the Books, and were modeled on regulations from other Human Libraries. For example, one of the Auburn Living Library’s rules is: ‘The Reader must return the Living Book in the same mental and physical condition as it is borrowed. Please do not cause damage to the Book, tear out or bend pages, spill food or drink over the Book or hurt his or her dignity in any other way. The Reader is responsible for taking care of and preserving the condition of the Book.’ ( Dreher & Mowbray, 2012).

•   The ‘Guidelines for Books’ document that was sent to Books when they were recruited (see Tip #8; Malhi *et al.*, 2023) also included advice for safely sharing their stories during the event. Recommendations included taking refreshment breaks, de-escalating tense discussions, and if necessary, ending the discussion. We also advised Books that they do not need to answer intrusive or rude questions from Readers, with one possible response being ‘Sorry, that particular story/page is not available.’

•   Event organizers should also be trained to recognize and intervene in potentially disruptive incidents where discussion between a Book and Reader has escalated to confrontation. Our recommended course of action to manage the situation would be to stop the session and ask the disruptive Reader to leave the event. The Book and remaining Readers may need a few minutes to regain their composure before proceeding with the session.

## Tip 10: Keep the Human Library event running smoothly and encourage participation

With the commencement of the Human Library event, all of the planning and work by organizers and Books comes to fruition. Our experience suggests that with sufficient advance preparation, the event itself often runs surprisingly smoothly. As mentioned in Tip #3, our in-person Human Library was scheduled with three reading sessions, with a 10-10-10 format: 10 minutes for Books to present their stories, 10 minutes for interactive ‘question and answer’, followed by 10 minutes for Readers to have some refreshments and transition to the next Book session. The entire event, from Reader check-in to concluding remarks, was completed within three hours.

Having a time-keeper and moderator provides attendees with a familiar sense of structure. In our case, the time-keeper alerted participants when Reading sessions were ending. The moderator announced the beginning and end of the event, counted down when Books should be wrapping up their sessions, gave out door prizes, and thanked the Books and event sponsors. In addition, we had a number of volunteers who maintained an orderly flow of Readers between sessions, answered any questions, and were available to handle any possible disruptive interactions (Tip #9).

The success of a Human Library event is also dependant on having lively conversations between Books and Readers. Therefore, it is important to encourage shy Readers to not only listen to a Book’s story, but also to pose questions or engage in the discussion. Both of the ‘Rules for Readers’ (Tip #9) and ‘Guidelines for Books’ (Tip #8) documents listed ‘ice-breaker’ questions to initiate or continue a conversation. For example, a Book could ask reticent Readers ‘Why did you choose my Book?’ or a Reader could ask a Book ‘What are the lessons you have learned from your experience?’

## Tip 11: Evaluate Human Library using feedback from Readers and Books

Human Libraries have been shown to provide a variety of tangible and intangible benefits for Readers, such as building compassion and empathy, fostering community engagement, and increasing information literacy ( Blizzard *et al.,* 2019). Even if your Human Library is not funded or is not research-oriented, it is still a good idea to collect data to assess the effectiveness of the event, including the benefits perceived by the attendees.

In our case, we used the event registration form to gather demographic information about Readers as well as their reasons for attending the event (Tip #7). After the session, we asked Readers about which Book session(s) they attended, the take-home messages that they found to be memorable, their satisfaction with the event, suggestions for improving future Human Libraries, and general comments about the event. We also included questions designed to compare pre-session and post-session attitudes towards rural medicine.

In addition, we encourage organizers to solicit feedback from the Books at the Human Library about their experiences and suggestions for improvement. This information could be collected through open-ended questions on a survey, or during one-on-one interviews with research personnel. Available literature shows that telling their story often has profound impact on a Book. Dobreski and Huang (2016) identify eight different categories of benefits for Books in Human Libraries, including altruism (helping others, teaching), making connections with diverse Readers, and self-focused benefits (learning from Readers, self-expression, personal enjoyment, etc.). For some Books, participating in a Human Library allows them to self-reflect and re-evaluate their narrative ( Kudo *et al.*, 2011) or develop plans for ‘next steps’ in their own personal journey ( Giesler & Juarez, 2019).

## Tip 12: Send tokens of appreciation to Books

The individuals who have volunteered to be Books are the heart of any Human Library event. Through the retelling of their stories, Books often make themselves very vulnerable. It may take tremendous strength for them to share deeply personal, and perhaps even traumatic experiences, with strangers. Human Library Books often exemplify the range of human struggles and triumphs, and their stories can be sources of inspiration to others. Therefore, it is important for Human Library event organizers to show appreciation and gratitude to the Books, both for their time and their courage in disclosing intimate stories.

On a pragmatic note, if you decide to hold more Human Library events in the future, you may want call upon the same Books to participate. Sincere appreciation from event organizers and gestures of goodwill can sustain connections with Books so that they remain in the Human Library ‘catalogue’. We recommend providing Books with tokens of appreciation that are meaningful and where possible, personalized. For our in-person Human Library event, we presented Books with a small honorarium and a framed copy of their book jacket.

## Conclusion

The Human library was originally conceived as a method of confronting prejudice, but the approach has, over time, proved to be remarkably versatile. At our institution, the Human Library has been successfully deployed as a way to provide perspectives and create discussion about rural medicine. Groups at other institutions may consider using the Human Library approach to address a wide variety of challenging subjects where the potential Readers could be biased or lack experience.

A Human Library differs greatly from traditional ways of delivering medical education curriculum, but its very novelty and emphasis on human connection may spark increased attention and deeper processing of messages in the audience. We note that medical school groups preparing to run Human Library events need to consider unique issues of safety and logistics that may not be part of the planning for general curriculum, but are critical for the success of the Human Library.

We recommend that event organizers consider the issues raised in the tips in order to support a successful Human Library. In addition, we encourage Human Library organizers to imbed scholarship into various facets of the event as a way to further explore the possibilities of this innovative approach within medical education.

## Ethics

Ethical approval for this project was provided by the University of Calgary Conjoint Health Research Ethics Board (Ethics ID REB19-0943).

## Data Availability

No underlying data are associated with this article. Open Science Framework: Practical Tips for using a Human Library approach in medical education. https://doi.org/10.17605/OSF.IO/AENK7 ( Malhi *et al.*, 2023). This project contains the following extended data: Human Library - Guidelines for Books.pdf Human Library - Rules for Readers.pdf Data are available under the terms of the Creative Commons Zero "No rights reserved" data waiver (CC0 1.0 Public domain dedication).
