# Supplementary material for: Practical Tips for using a Human Library approach In medical education
Source: MedEdPublish (2016). 2023 Oct 5;13:208. [Version 1] doi: 10.12688/mep.19746.1 (PMC10873544; doi:10.12688/mep.19746.1)
Supplement: Peer review history

# Reviewer report: Reviewer response for version 1

**DOI:** 10.21956/mep.21156.r35875

**Published:** 2024-02-16

**Authors:** Kirsty Pope

**Affiliations:** 1 Monash University, Clayton, Victoria, Australia

Thank you for giving me the opportunity to review this article. I have experience of running similar events and found the 12 tips very much reflected the practicalities we have also encountered. 

 Tip 1. The mention of gaining ethics in this section makes it sound like this is needed for all Human Library events. You mention in Tip 11 that this was needed as part of the evaluation process so I think it would be useful to briefly note this, either in the introduction or within Tip 1. As it's written, readers may think that they will need ethical approval to run a Human Library. 

 Tip 2. You mention that organizers require technological experience when the sessions are run online. It is probably worth adding here (or in Tip 8) that the Books may also need support with accessing the online platform. 

 You make the point that face-to-face was more attractive to attendees (this is also our experience) and support this point by saying how many people registered for each one. I'm wondering though how much this is really a good indicator as, from what you've said, the second event was advertised to a larger cohort of people. 

 Tip 3. Door prizes and draws for gift cards. It would be interesting to note somewhere in the article if you think these are necessary. This might be seen as a prohibitive factor if others don't have access to such resources. 

 Grammatical note: 'Having multiple Readers per Book is practical as it allows [plural needed] more people to hear the story' 

 Tip 7. It would be useful to know how many Books the Readers read during the session. In our session for example, Readers meet with 2 Books. 

 Tip 9. This is such an important part of the event and it's great to see the resources you provide to both Books and Readers. I think it would be useful to make it clear in this section that Readers are asked to follow confidentiality rules so they mustn't discuss the story outside of the Human Library unless given permission by the Book. 

 Tip 12. Grammatical note: 'you may want TO call upon the same...'

If evidence from practice is presented, are all the underlying source data available to ensure full reproducibility?

Yes

Is the topic of the practical tips discussed accurately in the context of the current literature

Yes

Are all factual statements correct and adequately supported by citations?

Yes

Are the conclusions drawn balanced and justified on the basis of the presented arguments?

Yes

Are arguments sufficiently supported by evidence from the published literature and/or the authors’ practice?

Yes

Reviewer Expertise:

Experience of running and evaluating Human Libraries.

I confirm that I have read this submission and believe that I have an appropriate level of expertise to confirm that it is of an acceptable scientific standard.

# Reviewer report: Reviewer response for version 1

**DOI:** 10.21956/mep.21156.r35872

**Published:** 2024-02-16

**Authors:** Hei Ting Wong

**Affiliations:** 1 National University of Singapore, Singapore, Singapore

Thank you for hosting and studying human library events, which are definitely a meaningful way to eliminate prejudices in society. I believe this paper has the potential to benefit human library practices and (medical) education if improvements can be made in two areas:

1. Engage and learn from more relevant research work. I suggest the authors should refer to more previous human library literature in educational settings (which actually dominated human library research). It is recommended to refer to published work rather than conference proceedings to strengthen the paper's foundation. I am sharing with the authors my co-authored paper: Lam *et al.* (2023 ^1^). I am *not* telling the authors to cite it. It is a review paper on human library literature and I hope the authors can locate some relevant references.
2. The authors' human library experience is unclear. As the authors hosted two human libraries, which were mentioned at the forefront, they should be valuable resources in offering the tips. Your practical tips should be derived from your experience. Also, the lack of relevant references reduced the validity of these suggestions. Occasionally, some statements are not well supported. For example, in the section "Tip 2," the authors stated, "In our experience, a face-to-face Human Library is more attractive to attendees" compared with a virtual meeting. However, other elements would affect participation rate other than simply "Zoom fatigue," such as inadequate promotion, short promotional time, and/or unappealing topics, for which the data was not available. 

If evidence from practice is presented, are all the underlying source data available to ensure full reproducibility?

Partly

Is the topic of the practical tips discussed accurately in the context of the current literature

Yes

Are all factual statements correct and adequately supported by citations?

Partly

Are the conclusions drawn balanced and justified on the basis of the presented arguments?

Yes

Are arguments sufficiently supported by evidence from the published literature and/or the authors’ practice?

Partly

Reviewer Expertise:

sound, voice, cultural, and media studies: meanings of voice in mediated contexts including the representation of vulnerable groups

I confirm that I have read this submission and believe that I have an appropriate level of expertise to confirm that it is of an acceptable scientific standard, however I have significant reservations, as outlined above.

## References

[1] : A Systematic Narrative Review of Implementation, Processes, and Outcomes of Human Library. *Int J Environ Res Public Health* .2023;20(3) : 10.3390/ijerph20032485 10.3390/ijerph20032485 36767851 PMC9915943

# Reviewer report: Reviewer response for version 1

**DOI:** 10.21956/mep.21156.r35002

**Published:** 2023-10-30

**Authors:** Lynsay Crawford

**Affiliations:** 1 University of Glasgow, Glasgow, Scotland, UK

The Human Library is a registered trademark of the Danish organization - the Human Library. – that you mention in the introduction. I don’t think you can call your event ‘Human Library’ events without it being an event linked to and run by them – you may need to change your nomenclature to one that states this is an event using 'people with lived experience'. The Human Library are very protective of their brand and I think you will need to change this.

 The rest of the article was well written and the practical tips were appropriate, well explained and covered all themes, based on my experience of running similar events.

If evidence from practice is presented, are all the underlying source data available to ensure full reproducibility?

Yes

Is the topic of the practical tips discussed accurately in the context of the current literature

Yes

Are all factual statements correct and adequately supported by citations?

Yes

Are the conclusions drawn balanced and justified on the basis of the presented arguments?

Yes

Are arguments sufficiently supported by evidence from the published literature and/or the authors’ practice?

Yes

Reviewer Expertise:

medical education, general practice,

I confirm that I have read this submission and believe that I have an appropriate level of expertise to confirm that it is of an acceptable scientific standard, however I have significant reservations, as outlined above.
